# Another Good Man Gone Wrong

**Published:** 1893-02

**Authors:** 


**Another Good Man Gone Wrong.**—Dr. R. H. Jones, late of Washington county, Texas, who killed Col. G. W. Veal in Dallas, last fall, as alleged, for raping, twenty years ago, the woman who afterwards became and is now Mrs. R. H. Jones, and who was on the 3d of February inst. convicted of murder in the first degree and given a life sentence, was the second President of the Texas State Medical Association, and the first First Vice-President. On the organization of the Association at Houston, in 1869, Dr. T. J. Heard, of Galveston, was elected President, and Dr. R. H. Jones, First Vice-President; the next year Jones was made President, and presided in 1870, at Houston.

This is bad enough, but he has not been selling any "Hepatic Pi[illegible]," (dose 1 to 3).

